# Design, methodology, and preliminary results of the non-human primates eye study

**DOI:** 10.1186/s12886-023-02796-6

**Published:** 2023-02-07

**Authors:** Jian Wu, Wei Liu, Sirui Zhu, Hongyi Liu, Kezhe Chen, Yingting Zhu, Zhidong Li, Chenlong Yang, Lijie Pan, Ruyue Li, Caixia Lin, Jiaxin Tian, Jiaoyan Ren, Liangzhi Xu, Hanxiang Yu, Fagao Luo, Zhiwei Huang, Wenru Su, Ningli Wang, Yehong Zhuo

**Affiliations:** 1 Beijing Ophthalmology & Visual Sciences Key Laboratory, Beijing Institute of Ophthalmology, Beijing Tongren Eye Center, Beijing Tongren Hospital, Capital Medical University, No. 1 Dong Jiao Min Xiang Street, Dongcheng District, Beijing, 100730 China; 2 State Key Laboratory of Ophthalmology, Zhongshan Ophthalmic Center, Guangdong Provincial Key Laboratory of Ophthalmology and Visual Science, Sun Yat-Sen University, Guangzhou, 510060 China; 3 School of Food Sciences and Engineering, South China University of Technology, Guangzhou, 510641 China; 4 Xuanwu Hospital, Capital Medical University, Beijing, 100053 China; 5 Department of Neurosurgery, Peking University Third Hospital, Haidian District, Beijing, China; 6 Guangzhou Huazhen Biosciences, Guangzhou, 510900 China

**Keywords:** Non-human primates, Eye study, Methodology, Ocular parameters

## Abstract

**Purpose:**

To describe the normative profile of ophthalmic parameters in a healthy cynomolgus monkey colony, and to identify the characteristic of the spontaneous ocular disease non-human primates (NHP) models.

**Methods:**

The NHP eye study was a cross-sectional on-site ocular examination with about 1,000 macaques held in Guangdong Province, southeastern China. The NHPs (*Macaca fascicularis,* cynomolgus) in this study included middle-aged individuals with a high prevalence of the ocular disease. The NHP eye study (NHPES) performed the information including systematic data and ocular data. Ocular examination included measurement of intraocular pressure (IOP), anterior segment- optical coherence tomography (OCT), slit-lamp examination, fundus photography, autorefraction, electroretinography, etc. Ocular diseases included measurement of refractive error, anisometropia, cataract, pterygium, etc.

**Results:**

A total of 1148 subjects were included and completed the ocular examination. The average age was 16.4 ± 4.93 years. Compared to the male participants, the females in the NHPES had shorter axial length and the mean Average retinal nerve fiber layer (RNFL) thickness (except for the nasal quadrants). The mean IOP, anterior chamber depth, lens thickness, axial length, central corneal thickness, choroid thickness and other parameters were similar in each group.

**Conclusion:**

The NHPES is a unique and high-quality study, this is the first large macaque monkey cohort study focusing on ocular assessment along with comprehensive evaluation. Results from the NHPES will provide important information about the normal range of ophthalmic measurements in NHP.

**Supplementary Information:**

The online version contains supplementary material available at 10.1186/s12886-023-02796-6.

## Introduction

It was estimated that 895 million people with vision impairment worldwide, of whom 61 million may suffer from blindness by 2050 [1]. About 65% of individuals with mild to severe visual impairment are over 50 years old [2], and aging populations face an increasing burden of ocular diseases, resulting in substantial health and economic burdens. Glaucoma, age-related macular degeneration, diabetic retinopathy, high myopia, cataracts are the most common aging-related ocular diseases [3–7], resulting in irreversible blindness worldwide. It is critical to identify the biomarkers and mechanism of visual impairment to prevent the occurrence of optic nerve degeneration and macular disease among middle-aged or older individuals through clinical and basic research.

To clarify the pathogenesis of ocular diseases, several animal models were developed to study the disease process [8–10]. Different from small animals like mice, non-human primates (NHP) have more human-resemble eye structures, such as the macula structure, and vitreous volumes. Meanwhile, the macaques share more than 90% DNA sequence and highly conserved protein sequences with humans [11], with the substantial similarities in anatomy, physiology and genetics [12] In addition, using invasive methods and novelty treatment in NHP studies may complement those in human volunteers and patients. Therefore, NHPs were regarded as an attractive model for providing new exploration directions and practical basis for human visual biology and ocular disease in preclinical studies [11].

With the increasing application of the NHP experimental model using to ocular research, it is truly needed to establish a preclinical evaluation system and standardized procedures for NHP ophthalmic examination. Meanwhile, considering the differences between NHP and human eye biological parameters, a standardized database of ophthalmic parameters in healthy NHP populations should be established for the disease model evaluation, a thorough understanding of the normal range of ophthalmic biological structure and retinal anatomical parameters in NHPs is critical for laying the foundation for preclinical visual science research [13]. Unlike the numerous human ocular epidemiological studies have been performed worldwide the previous NHP eye studies with small sample size and unrepresentative results [14, 15], there was a scarcity on a large-scale ophthalmic epidemiological study of NHPs to provide a reference range for normal values of ophthalmic parameters and reporting the screening information.

We held the NHP eye study for describing the normative profile of ophthalmic parameters in a healthy cynomolgus monkey colony, which might be helpful to provide criteria for using NHP experimental models. Meanwhile, since the NHP experimental model may have distinct pathophysiology from the spontaneously developed disease, we attempt to discover NHP spontaneous ocular disease models using for further investigated the pathogenesis of the ocular disease. Furthermore, the NHPS assist the preclinical research to identify the etiology and biomarkers of visual impairment in human ocular disease. In this report, we describe the objective, study design, baseline characteristics, and strengths and potential limitations of the NHP Eye Study (NHPES).

## Methods

### Study design

**Fig. 1:**
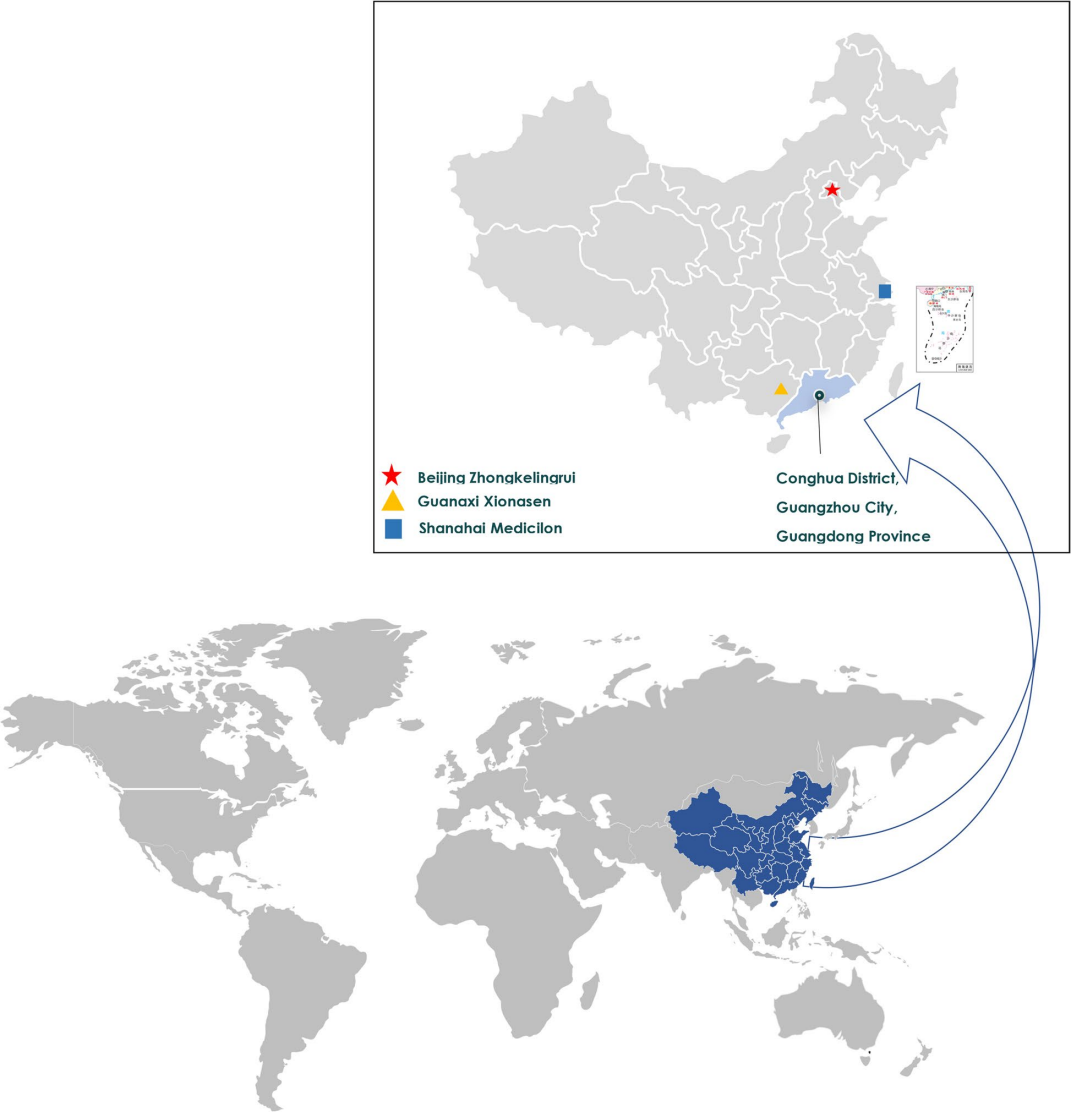
The location of non-human primates eye study

The NHP eye study (NHPES) was a cross-sectional on-site ocular examination with more than 1,000 macaques held in Guangdong Province, southeastern China. This study was performed from 2021 to 2022, the location was illustrated in Fig. 1. The NHPES was designed to describe the normative ocular parameters distribution in NHPs colony, discovered, and further displayed the characteristics of the spontaneously developed ocular disease NHPs using the digitization of image data.

This study complied with the National Institutes of Health Guide for the Care and the guidelines of the Association for Research of Vision and Ophthalmology for the Use of Animal in Ophthalmic and Vision Research. The study was approved by the Ethical Committee of the Guangzhou Huazhen Biosciences Company (Ethics Number: 2020–168) and Zhongshan Ophthalmic Center (Permit Number: SYXK (YUE) 2018–0189).

### Animals

All NHPs (*Macaca fascicularis,* cynomolgus) included in this study were from Huazhen Biosciences(HZ-Bio) company and were treated following the ARVO Statement. HZ-Bio is a subsidiary of Huazhen Laboratory Animal Breeding Center, which is a fully Association for Assessment and Accreditation of Laboratory Animal Care (AAALAC)-accredited facility permitted to perform laboratory animal research in China.

HZ-Bio is a company that specialized in NHP research, with the mission to provide research models and services to the biomedical research community and bio-pharmaceutical industry. All macacas were housed in air-conditioned rooms at temperatures of 16 °C-26°C with a relative humidity of 40-70% and a 12 h light/12 h dark cycle in a climate-controlled room. Free access to drinking water and commercial primate diet (12% calories from fat, 18% calories from protein, and 70% calories from carbohydrates at 200 to 300 g/d), fresh fruits, and additional supplements were also provided on a daily basis. Meanwhile, entertainment and welfare including toys and music were also provided. In addition, veterinarians with more than 20 years of experience were 24-h monitoring the healthy status of cynomolgus.

### Specific aims

1. To establish a normative database of cynomolgus monkeys, and describe the normal range of the ocular parameters.
2. In search of the spontaneously ocular disease models in NHPs used in the following basic and preclinical studies (exploring the pathogenesis, novelty treatment such as gene therapy and new biopharmaceuticals).
3. To describe ophthalmic characteristics of the NHPs defined by the spontaneously developed ocular diseases model.
4. To provide a standard reference range for the experimental ophthalmic disease model in cynomolgus monkeys.
5. To establish a preclinical evaluation system and standard operating procedure for NHP in ophthalmic examination

### Sample size considerations

We used the following calculation formula to estimate the sample size: n = ${u}_{a/2}^{2}$ × p × (1-p)/d2. Considering a confidence interval of 95% (bilateral), ${u}_{a/2}^{2}$of 1.96. In the formula, d represented the difference between the prevalence of the sample and the population, here we set it as 1%. Based on the previous studies, the prevalence of the main ocular diseases was estimated to be over 2% [3, 16–19], as the p value in the formula was set as 2.5% in this study. Based on the data above and assuming a final response rate of 85%, about 1,100 subjects would be recruited for this study. The target subject included middle-aged individuals with a high prevalence of the ocular disease. Aged over 10 years in good healthy state were included in the examinations.

### Examination procedures

**Fig. 2:**
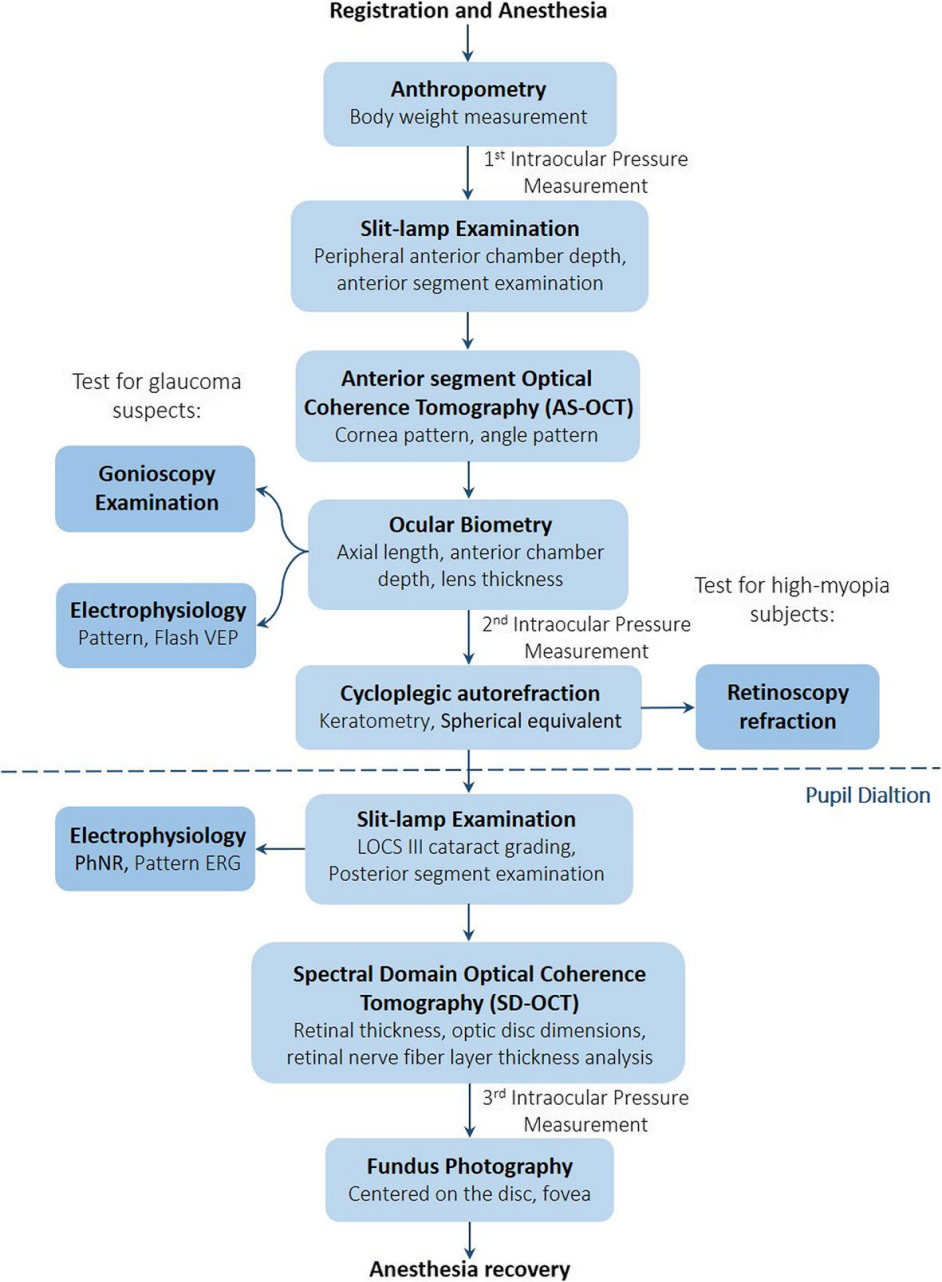
The procedure of ocular examination for macaques in non-human primates eye study

All subjects were anesthetized by intramuscular injection of Zoletil (4 mg/kg body weight, Virbac) mixed with xylazine/ketamine 0.2 mg/kg (Sumianxin, Shengda Animal Medicine), and underwent all examinations in 60 to 120 min according to the standardized protocols. The NHPES performed the information including systematic data (sex, age, weight), and ocular data (refractive state; intraocular pressure (IOP); slit-lamp examination; fundus photography; AL-100 examination; optical coherence tomography (OCT) image, etc.). The anesthesia dose was within safety range while veterinarians took care of the examination site during the whole process. The procedures were outlined in Fig. 2.

1. Registration: At the beginning of the NHPES, each subject was encoded and registered according to the specific identification brand. The systematic data including sex, date of birth, age, and history of surgery were recorded. The body weight was measured according to a standardized protocol by a certified veterinarian.
2. IOP Measurements: After being anesthesia the technician stabilized the monkey in a flatting position and kept its head in a primary gaze state. An eyelid speculum was used to immobilize the eyelids, while the IOP measurements were conducted by an experienced technician using an Icare tonometer (TA01i; Icare, Helsinki, Finland) collimating the center of the pupil. The instrument automatically collected and displayed the value of six average measurements. The IOP measurements were excluded if the difference between each measurement exceeded 3 mmHg and we obtained six measurements in three series. All subjects measured the IOP at the beginning, middle, and end of anesthesia, three times measurements were obtained finally.
3. Anterior Segment-OCT Measurements: The anterior chamber structure was scanned using anterior segment OCT (Heidelberg Engineering GmbH, Heidelberg) before dilation. Each scan included the scleral spur and iris recess. Both cornea and angle patterns were conducted in each subjects to determine the central corneal thickness (CCT), anterior chamber width (ACW), and angle parameters (angle opening distance [AOD], angle recess area [ARA], trabecular-iris space area, and trabecular-iris angle [TIA]) analyzed using the manufacturer provided software.
4. Dilation: Macaques were properly situated in a headrest position and pupils were dilated by the application using one drop of 0.5% tropicamide phenylephrine hydrochloride (Mydrin P, Santen, Osaka, Japan) in both eyes after anterior segment examination. A self-retaining eyelid speculum was placed to enable the examination until the pupil diameter reached ≥ 6 mm.
5. Slit-lamp Examination: Examinations were performed by experienced ophthalmologists after anesthesia (described above) using slit-lamp biomicroscopy (TOPCON Slit-lamp SL-D701) to evaluate the overall structures of the anterior segment, including eyelids, cornea, anterior chamber, lens, iris, and related structures. The slit-lamp beam held a width of 0.3 mm and a height of 9.0 mm for the lens photographs, and the angle between the slit-lamp beam and the sagittal axis was set as 45°. Initial examination confirmed the anterior segment diseases such as pterygium and keratitis, the peripheral anterior chamber depth was evaluated using the method of Van Herick [20]. The cataract diagnosis used the Lens Opacities Classification System III (LOCS-III) as a reference [21]. All subjects were checked the right eye first followed by the left eye.
6. Fundus Photography: Experienced ophthalmic technicians used a digital fundus scope(APS-BER Fundus Camera & FFA model, AITOMU) to obtain the fundus photos after adjusting to ensure the image was focused clearly. Each subject was taken with at least two high-quality fundus photographs following the operation rules, bilateral retinal 45-degree images of the optic nerve (centered on the disc), and the macula (centered on the fovea). Both eyes of each subject were well taken. Fundus photographs were graded preliminarily for the level of fundus lesions by two graders.
7. Optic Nerve Head (ONH) Parameter Measurements: The examinations were performed after pupil dilation using Heidelberg Spectral-domain OCT (Heidelberg Engineering GmbH, Heidelberg, Germany). The images of ONH scans were obtained by circular scans. The location of the aiming circle was adjusted by the experienced operators to match the ONH, and the 24 radial scans (scans ranging from 1.3 to 4.9 mm) were well-conducted covering a 3.4 mm diameter circle that included ONH surroundings structure. ImageJ software (National Institutes of Health, https://imagej.nih.gov/ij/) was used to measure the LC parameters (Bruch's membrane opening, LC depth, posterior LC surface depth, anterior laminar insertion depth, LC thickness, LC curve index).
8. Retinal Nerve Fiber Layer (RNFL) Measurements: The RNFL thickness was assessed automatically by the Heidelberg OCT software into six sectors. Included temporal (T; 315° to 45°), temporal-superior (TS; 45° to 90°), temporal-inferior (TI; 90° to 135°), nasal (N; 135° to 225°), nasal-superior (NS; 225° to 270°), and nasal-inferior (NI; 270° to 315°), respectively. The means of RNFL thickness were calculated by averaging the thickness values of 360° measures.
9. Retinal Thickness (RT) Measurements: RT scan was carried out between the internal limiting membrane (ILM) and the edge defined by the mean value of the maximum reflectance of the RPE [retina 9]. The built-in software automatically generates the RT map by a common center of three concentric circles was located in the macular fovea, and the diameters were 1 mm (innermost ring), 3 mm (inner ring), and 6 mm (outer ring), and was divided into 9 sectors (central macular areas, above the inner ring, below the inner ring, nasal side of the inner ring, temporal side of the inner ring, above the outer ring, below the outer ring, nasal side of the outer ring and temporal side of the outer ring) according to the definition of Early Treatment Diabetic Retinopathy Study (ETDRS). The signal strength index (SSI) less than 15 dB (as recommended by the manufacturer) was excluded further from the analysis.
10. A-scan Ultrasonography: Ocular biometry parameters were acquired by using the A-scan ultrasonography (Tomey AL-4000, Tomey, Nagoya, Japan). Anterior chamber depth, lens thickness and axial length were examined with a transducer frequency of 10 MHz applied to execute measurement. The upper eyelid was manually retracted with an eyelid speculum was placed to facilitate. Directly put the ultrasound probe with an integrated red fixation light at the central cornea in a contact mode, the measurement will automatically start if the system detects a sufficient sound echo. Data along with standard ultrasonic waveforms of cornea, anterior and posterior surface of lens and retina as well as standard deviation less than 0.13 mm were acceptable and recorded the ACD, LT, AL value respectively.
11. Autorefraction Measurements: Refractive status was measured using an autorefractor (Fario FXR-710), with the combination function of optometer and keratometer. Cycloplegia was administered one drop 1% tropicamide (Sandoz) every 5-min interval for twice. A third drop would be administered if pupillary light reflex was still present or the pupil size was less than 6.0 mm 30 min after the last drop. Spherical refraction (SR), cylinder refraction (CR) and corneal radius of curvature (CRC) were repetitively measured for three times, the mean of SR, CR, SE and CRC were calculated to analyze.
12. Retinoscopy Refraction: Subjective Refraction was performed by one experienced technician and one optometrist. Cycloplegic retinoscopy was used to determine the spherical and cylindrical components on subjects with uncorrected visual acuity less than 20/40 in either eye after pupil dilation. To avoid observer bias, the optometrist who performed retinoscopy was blinded to the results of autorefractors. An average of three measurements was acquired.
13. Electroretinography (ERG): ERG technology (Roland consult® RETImap Model 520 ERG, Germany) was used to quantify neuroretinal, retinal ganglion cells and visual pathway function. Active electrodes were placed into the eyelid, references electrode was placed at both sides under the skin and the ground electrode was placed near the tail of the subject. Electrode impedance was acceptable with the difference less than 2 KΩ, and the pattern VEP and flash VEP were conducted after the electrodes were proper placed. After both eye were full dilatation using topical eye drops (previously mentioned), the pattern ERG and photopic negative response (PhNR) test was performed according to the International Society for Clinical Electrophysiology of Vision (ISCEV) standard protocol.

### Quality control and data management

Quality control were conducted throughout the study procedures to ensure that the accuracy and completeness of the final dataset. The ophthalmologists, technicians, and assistant staff were trained to understand the aims of this study, the standardized examination procedures, and diagnostic criteria. A pilot study with approximately 100 subjects enrolled was conducted to ensure data consistency by determining the repeatability of the examination and diagnosis by different examiners. An experienced ophthalmologist (JW) and investigators supervised the field site to ensure and monitor the standardized procedures.

Initial data were collected with a combination of paper and digital formats. Ophthalmic examination records, printouts (eg, refractive parameters) and OCT reports were compiled into specific case report forms labeled with each subject’s unique study ID. Clinical examination data forms were reviewed for accuracy and completeness before input to electronic format. Imaging data including fundus photo, OCT image and lens photographs, were retrieved directly from ocular instruments and stored in the PC and backup in the mobile disk. Initial data were collected with a combination of paper and electronic formats, paper data were input by double entry and validation.

### Primary outcome measures

Refractive error and anisometropia: The refractive error was referenced by spherical equivalent (SE). Myopia and hyperopia defined as SE ≤  − 0.5 D and ≥  + 0.5 D (also alternatively as ≤  − 1.0 D and ≥  + 1.0 D) respectively. Astigmatism was defined as minus cylinders ≤  − 0.5 D or ≤  − 1.0 D. Anisometropia was referred to the difference from binocular SE more than 1.0 D or 2.0 D.

Cataract: The lens opacities classification system (LOCS) III [21] was used to evaluate the severity of lens opacity in four major characteristics: nuclear opalescence (NO), nuclear color (NC), cortical (C), and posterior subcapsular (P). Cataract was defined based on the lens images using slit-lamp reference to standard grading photos, and diagnosis as significant nuclear, cortical, or posterior subcapsular cataract.

Pterygium: The pterygium was defined under the slit-lamp microscope as an extension of conjunctiva onto the clear cornea without an alternative explanation such as ocular trauma. Pterygium was graded in grade 1 (transparent), grade 2 (intermediate) and grade 3 (opaque) based on relative transparency of pterygium tissue.

Posterior segment eye diseases: Age-related macular degeneration (AMD) was graded by fundus photo using the Wisconsin AMD classification system [22]. Glaucoma was defined using the recommendations of the Association of International Glaucoma Societies [23]. Meanwhile, myopic retinopathy was categorized by the International Photographic Classification and Grading System. For the diabetic retinopathy diagnosis, the grading protocols was according to the Early Treatment Diabetic Retinopathy Study (ETDRS) adaptation of the modified Arlie House classification of DR criteria [24]. And non-arteritic anterior ischemic optic neuropathy (NAION) diagnosis criteria was defined as a small optic disc (a cup/disc diameter ratio of 0.3 or less), with segmental pallor or segmental loss of retinal nerve fiber layer [25]. All images and relevant data were reviewed by two well-trained ophthalmologists, a third senior specialist will determine the final diagnosis when appear the consensus on diagnosis.

### Statistical analysis

Statistical analysis was performed using statistical software (SPSS 23.0 and SAS 9.4, SAS Institute, Cary, NC, USA). Data was performed as the mean ± standard deviation (SD) for continuous variables and number (%) for categorical variables. An independent t-test or analysis of variance (ANOVA) was applied to compare the differences between groups in normally distributed quantitative data. A Wilcoxon or Kruskal–Wallis test was used for non-normal data. The Chi-squared test was use to evaluate categorical variables. The statistical strength of correlations was described using odds ratio (OR), confidence intervals (CI, 95%) were also presented. *P* < 0.05 (two-tailed) were considered as statistically significant.

## Results

During the period from July 2021 to June 2022, a total of 1148 subjects were included and completed the ocular examination (Fig. 2). Number 1029 finally took part in the study and the response rate was 89.6%

All participants were Macaca fascicularis (cynomolgus) species. Of the excluded-subjects, about 60% were pregnancy, 25% were older than 25 years, and 15% with systematic disease were not suitable for anesthesia. Supplementary Fig. 1 provides the complete details on selection of the study subjects. The systematic characteristics of including subjects were shown in Table 1. The majority of the included subjects were female (52.1%). The average age was 16.4 ± 4.93 years (range 2 years to 26 years) (Table 1).

**Table 1:** General characteristics of the included subjects in the non-human primate eye study

Characteristics	Total	Female no. (%)	Male no. (%)	*P* value
N	1029	536(52.1%)	493(47.9%)	0.016
Age (SD), years	16.4 ± 4.93	18.33 ± 3.87	14.23 ± 5.06	< 0.001
Age distribution				
1–5	47(4.59%)	14(29.79%)	33(70.21%)	0.166
6–10	97(9.46%)	11(11.34%)	86(88.66%)	0.930
11–15	211(20.59%)	70(33.18%)	141(66.82%)	0.031
16–20	462(45.07%)	262(56.71%)	200(43.29%)	< 0.001
21–26	208(20.29%)	175(84.13%)	33(15.87%)	0.072
Weight (kg)	4.52 ± 1.23	4.10 ± 0.84	6.12 ± 1.16	0.007

All data were shown as M ± SD or n (%)

**Fig. 3:**
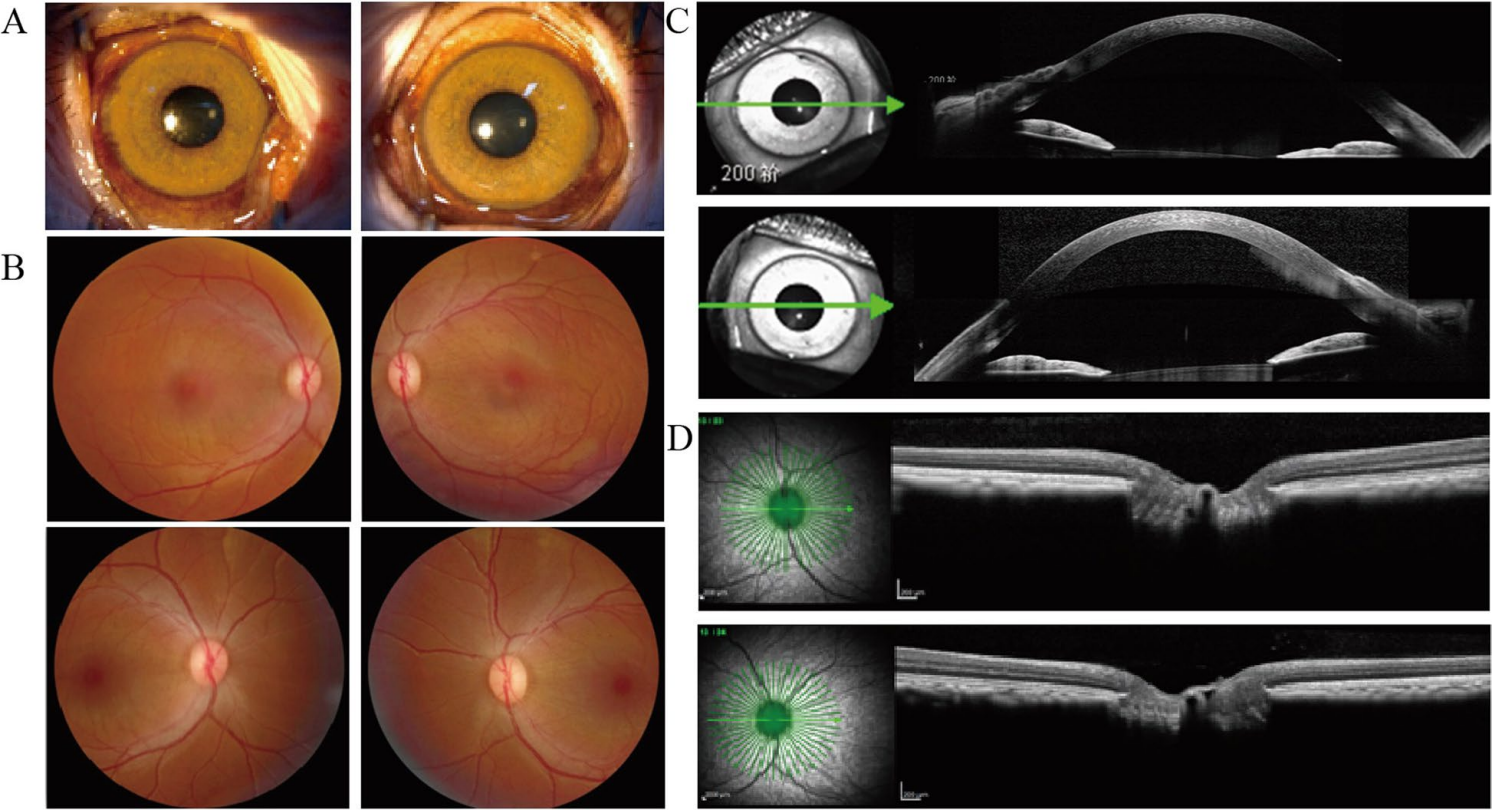
The photos of a health female monkey aged 19, including silt-limp photos (**A**), colorful fundus photos (**B**), anterior segment OCT photos (**C**) and posterior segments OCT photos (**D**)

Table 2 showed the general features of majority ophthalmic parameters, and presented the primary results of the physical measurements for the male and female subjects in both eyes. We measured the parameters of the eyes of monkeys with different genders. Compared to the male participants, the females in the NHPES had shorter axial length which may related to the weight measurement (Table 2). The mean IOP, Anterior chamber Depth, Anterior chamber depth, Lens Thickness (LT), axial length (AL), central corneal thickness (CCT), choroid thickness (CT) and other parameters were similar in each group. However, the mean spherical equivalent (SE) of OS (-1.47 ± 3.61 diopters, Female; -1.62 ± 3.68 diopters, Male) were higher than OD (-0.55 ± 3.63 diopters, Female; -0.81 ± 3.51 diopters, Male). As for the mean Average RNFL thickness. The results were similar except for the nasal quadrants, in the nasal quadrant, the mean value of male monkeys (77.43 ± 34.93 OD; 78.76 ± 40.29, OS) was significantly higher than that of female (58.07 ± 12.65, OD; 78.76 ± 40.29, OS). In Table 2, according to comparison of female’s OD and OS, IOP 2^nd^,3^rd^, IOP, SE, corneal radius of curvature, ACD, AXL, CCT, average RNFL thickness have statistical significance. Male have similar trend expect SE, ACD, with LT and RNFL thickness have statistical significance. Comparing the females and males, IOP, corneal radius of curvature, ACD, LT, AXL, CT, RNFL thickness have statistical significance. Furthermore, we have compared several age-related ocular parameters of different age groups in supplementary table 2. The results of normality test are showed in supplementary table 3 and supplementary table 4. We meanwhile presented the photos from a healthy female monkey aged 19, including colorful fundus photos and OCT (both anterior and posterior segments) in Fig. 3.

**Table 2:** Ocular parameters of the included subjects in the non-human primate eye study

Characteristics	Female			Male			F/M OD t-P**
	OD	OS	t-P*	OD	OS	t-P*	
IOP (mmHg)							
1^st^	23.70 ± 3.51	23.49 ± 3.36	0.092	23.33 ± 3.71	23.03 ± 3.83	0.022	0.325
2^nd^	22.50 ± 5.70	22.18 ± 4.48	0.138	22.05 ± 4.48	21.73 ± 4.17	0.357	0.204
3^rd^	19.43 ± 4.23	18.97 ± 4.09	0.007	20.60 ± 3.67	20.22 ± 3.87	0.205	0.289
IOP(SD)	22.19 ± 3.57	21.75 ± 3.19	0.001	22.49 ± 3.12	21.99 ± 3.23	0.003	0.531
Spherical equivalent (D)	-1.33 ± 3.42	-1.47 ± 3.61	0.340	-1.61 ± 4.05	-1.48 ± 3.07	0.779	0.565
Corneal radius of curvature (um)	5.67 ± 0.22	5.66 ± 0.22	0.482	5.81 ± 0.20	5.80 ± 0.22	0.453	0.464
Anterior chamber depth (um)	3.25 ± 0.33	3.27 ± 0.31	0.138	3.24 ± 0.23	3.24 ± 0.22	0.985	0.006
Lens thickness (um)	3.37 ± 0.30	3.37 ± 0.29	0.764	3.50 ± 1.80	3.30 ± 0.25	0.307	0.038
Axial length (um)	18.69 ± 0.77	18.72 ± 0.84	0.371	19.01 ± 1.04	18.97 ± 0.99	0.342	0.001
CCT (um)	449.37 ± 41.89	456.39 ± 51.31	0.001	463.15 ± 33.01	467.90 ± 32.72	0.019	0.621
CT (um)	191.60 ± 29.31	192.25 ± 29.19	0.723	184.31 ± 48.08	185.51 ± 47.23	0.376	< 0.001
Average RNFL thickness (um)							
Superior	112.78 ± 14.93	110.78 ± 13.76	0.016	113.74 ± 18.18	117.40 ± 29.80	0.420	0.217
Inferior	128.84 ± 15.13	128.03 ± 17.07	0.394	120.39 ± 20.88	122.96 ± 26.19	0.423	< 0.001
Nasal	58.07 ± 12.65	55.34 ± 13.59	0.001	77.43 ± 34.93	78.76 ± 40.29	0.712	0.035
Temporal	71.26 ± 14.35	74.48 ± 13.45	0.001	70.61 ± 18.83	72.46 ± 23.62	0.347	0.565

All data were shown as M ± SD or n (%)

* SE* stands for Spherical equivalent, *IOP* stands for Intraocular pressure, *CCT* stands for central corneal thickness, *CT* stands for corneal thickness

P* is the result from comparison between female/male’s OD and OS and p** is from female’OD and male’s OD

## Discussion

Currently, we reported the rationale, methodology, and demographic characteristics data of systematic and ocular parameters in a NHP colony. The NHPES is a unique and high-quality study, to our knowledge, this is the first large macaque monkey cohort study focusing on ocular assessment along with comprehensive evaluation, to provide the normal range of ophthalmic measurements in NHP, to identify and describe the characteristic of the sponstaneous ocular disease NHP models.

In the long process of animal testing and the development of new drugs, monkeys play an indispensable role. Compared with rodents, primates have much similar biology to humans and are often modeled as monkeys with human diseases. However, laboratory monkeys are more demanding in terms of environment, space and climate, with more expensive price. On the choice of experimental monkey field, we gathered about Guangzhou HuaZhen biological (HZ-Bio) technology company, Beijing Zhongkelingrui biological technology company, Guangxi Weisen primate experimental animal breeding development company, and Shanghai Medicilon biological pharmaceutical company, as shown in Fig. 1. Considering that the growth environment of the monkey group is mainly in the southeast coastal area with warm climate, meanwhile our research needs a relatively large number of experimental monkeys, finally we chose Guangzhou HZ-Bio. There are several advantages, firstly, HZ-Bio located in the south of China, with the coastal area between 22°26 '– 23°56' north latitude and 112°57 '– 114°03' East longitude. It has a subtropical monsoon climate, warmer and rainy, and has rich vegetation, which is more suitable for the living habits of monkeys. Secondly, Hz-bio has the Association for Assessment and Accreditation of Laboratory Animal Care (AAALAC) accredited facility with over 32,000 quality rhesus monkeys (crabeater monkeys). Monkey cages are much more numerous, with more than 500 single cages and 20 group cages, as well as fully equipped LABS and surgical rooms. Thirdly, Hz-bio is dedicated to NHP research and has more than 20 years of experience in providing quality NHP animal models. Furthermore, Hz-bio is an expert in animal medicine and care.

Compared with the data of healthy monkey eyes in previous studies (Table 3), the difference in intraocular pressure is not significant. While lens thickness and AL are smaller, compared with the results of Kira H. Lin et al. [26]. For the Average RNFL thickness, our results were significantly higher than those of Corinne Maurice et al. [27], which may be due to their use of postmortem monkey eyes. Compared with the study with larger sample sizes, the RNFL thickness of monkeys was similar to that of normal adults in the Superior, Inferior, and Temporal quadrants, but the Nasal RNFL thickness of female monkeys was thinner (Alasil et al. 75.0 ± 14.0; Zangalli et al. 86.0 ± 13.8; Appukuttan et al. 79.7 ± 12.1;) [28–30]. It may be related to the thinning only in the nasal region (the thinnest RNFL region) obtained in the study on the influence of IOP on RNFL mentioned by Jordan Dwell et al. [31].

**Table 3:** Ocular parameters in previous non-human primate eye studies

study	Simple size	Age(year)	IOP(mmHg)	Lens thickness(um)	Axial length (um)	CCT(um)	Average RNFL thickness (um)						
							G	N	NS	TS	T	TI	NI
Lin et al. [27]	142	16.4 ± 7.5	18 ± 4	4.24 ± 0.53	20.06 ± 0.95	486 ± 38	NA						
Pasquale et al. [28]	722	14.4 ± 2.0	14.3 ± 2.5	NA	NA	NA	103.7 ± 5.5	61.4 ± 8.3	106.7 ± 14.1	148.0 ± 12.5	80.4 ± 10.6	167.6 ± 14.6	123.7 ± 19.1
							S		I		N		T
Maurice et al. [29]	17	NA	NA	NA	NA	NA	75.3 ± 26.5		69.4 ± 22.4		48.1 ± 15.0		49.2 ± 26.4
Zhang et al. [30]	3	NA	22.67 ± 4.04	NA	NA	NA	NA						
Ollivier et al. [31]	10	6.7 ± 1.4	14.8 ± 0.8	NA	NA	487 ± 20	NA						

All data were shown as M ± SD or n (%)

*IOP* stands for Intraocular pressure, *CCT* stands for central corneal thickness, *S* stands for superior sector, *I* stands for inferior sector, *N* stands for nasal sector, *T* stands for temporal sector, *NS* stands for nasal-superior sector, *TS* stands for temporal-superior sector, *TI* stands for temporal-inferior sector, *NI* stands for nasal-inferior sector

After comparing several population-based studies with our data, we found a similar prevalence of several eye diseases, including glaucoma, cataracts, AMD, and pterygium (Supplementary Table 1). Moreover, we found that the high prevalence of myopia in monkeys is higher than in humans, which may be caused by these factors. Firstly, nearly 10 monkeys were fed in a small room less than 5 m in length and width. Therefore, the monkeys had been looking at short-distance things essential for developing myopia. In addition, due to the monkeys' need for warmth, the ceiling had only one small window in the feeding room. As a result, most monkeys failed to receive full sunlight, which might be another cause of high myopia.

Our study have several advantages. The major strength of NHPES was including large sample size, with comprehensive ocular examinations, which provide the normative profile of ophthalmic parameters in cynomolgus monkey for assessing subtle changes in retinal anatomy in this species. Moreover, the captivity NHPs are considered to be good model animals for disease research since they grow up in a simplified environment. Furthermore, we used advanced instruments for measurement, such as multi-colour OCT and nonmydriatic colour fundus photography.

There are still several limitations to the NHPES study. Firstly, the selecting bias may exist for most including subjects were middle-to-old aged monkeys with a small age span, making it difficult to observe and interpret age-related changes in ophthalmic characteristics. Meanwhile, due to the objective facts of the monkey factory, there are still some deficiencies in sex and distribution. However, a young group study is planned for next year. Secondly, some of the data may be missing due to the large number of examination items in NHPES, which may leading to information biases due to data completeness. Thirdly, it was difficult to avoid the limitations of cross-sectional studies, such as the inability to establish a causal relationship. However, a follow-up study is being drafted and scheduled to be carried out in one years later.

Generally, we reported the detailed ocular features of a macaque monkeys colony under carefully ophthalmic examination. In the further study, we will dress out the normal value database in NHP for providing the reference when ocular disease model was built up. The salient characteristics of classic ocular disease will also be displayed. Overall, the data from the NHPES will give us unprecedented opportunity to insight the natural development and pathogenesis of ocular disease.

## Supplementary Information

**Additional file 1:** **Supplementary Table 1.** The prevalence of ocular diseases in NHPES [7, 13, 32–34]. **Supplementary Table 2.** The result of ocular parameters for gender in different age groups [35–46]. **Supplementary Table 3.** The ocular parameters of normality test in OD. **Supplementary Table 4.** The ocular parameters of normality test in OS. **Supplementary Fig. 1.** The flow chart of NHPES exclusion criteria and quality control (QC) process.

**Additional file 2: Appendix.** The non-human primates (NHP) eye study team.

## Data Availability

All data generated or analysed during this study are included in this published article [and its supplementary information files].
