# Supplementary material for: Design, methodology, and preliminary results of the non-human primates eye study
Source: BMC Ophthalmol. 2023 Feb 7;23:53. doi: 10.1186/s12886-023-02796-6 (PMC9903517; doi:10.1186/s12886-023-02796-6)

Supplementary table1. The prevalence of ocular diseases in NHPES.

| Ocular Disease | Prevalence in NHPES | Prevalence in human study |
| --- | --- | --- |
| Glaucoma | 0.94% | 1.00% [7] |
| High Myopia | 10.19% | 2.70% [44] |
| Cataracts | 28.44% | 20.80% [45] |
| AMD | 9.38% | 8.69% [46] |
| Pterygium | 1.41% | 1.30% [34] |

Supplementary table2. The result of ocular parameters for gender in different age groups.

| **Variables** | **Female** | | | |  | **Male** | | | |
| --- | --- | --- | --- | --- | --- | --- | --- | --- | --- |
|  | **＜15** | **15-20** | **＞20** | **P*** |  | **＜10** | **15-20** | **＞20** | **P**** |
| RNFL  S  I  N  T | 116.00±14.14  128.50±0.71  50.00±1.41  68.00±7.07 | 112.85±15.02  129.01±15.65  58.05±12.79  71.16±14.77 | 111.56±14.25  128.38±11.99  59.31±12.88  71.56±11.34 | 0.860  0.976  0.581  0.941 |  | 117.07±13.48  132.80±18.42  111.08±31.43  79.53±12.03 | 110.40±5.27  113.60±18.08  60.51±23.16  73.00±7.00 | 111  125.92±18.10  121  58 | 0.546  0.149  0.409  0.142 |
| ACD | 3.55±0.38 | 3.25±0.33 | 3.23±0.34 | 0.271 |  | 3.27±0.20 | 3.21±0.21 | 3.27±0.33 | 0.547 |
| Lens thickness | 3.05±0.17 | 3.34±0.28 | 3.60±0.29 | ＜0.001 |  | 3.18±0.24 | 3.80±2.62 | 3.39±0.29 | 0.363 |
| AXL | 18.52±0.52 | 18.66±0.76 | 18.76±0.76 | 0.712 |  | 18.90±1.07 | 19.04±1.02 | 19.14±1.09 | 0.764 |
| CCT | 480.00±11.31 | 449.96±43.25 | 445.53±33.15 | 0.505 |  | 463.95±30.68 | 463.57±37.41 | 460.62±24.33 | 0.955 |
| CT | 202.00 | 193.79±28.19 | 182.62±33.06 | 0.227 |  | 149.42±46.15 | 189.82±44.12 | 209.92±45.57 | 0.001 |

Note: P* is the result of ANOVA for female different age ranges and p** is for male. All data were shown as M± SD or n (%). RNFL stands for retinal nerve fiber layer, ACD stands for Anterior chamber depth, Axial length stands for Axial length, CCT stands for Central corneal thickness, CT stands for Choroidal thickness

Supplementary table 3. The ocular parameters of **normality test in OD**

|  | [sample size](javascript:;) | [mean](javascript:;) | | SD | [skewness](javascript:;) | | [kurtosis](javascript:;) | kolmogorov-smirnov test | |  | Shapiro-Wilk W test | |
| --- | --- | --- | --- | --- | --- | --- | --- | --- | --- | --- | --- | --- |
|  |  |  |  |  |  |  |  | statistic | P |  | statistic | P |
| IOP (mmHg) |  | |  | | | | | | | | | |
| 1st | 516 | 23.58 | | 3.58 | -0.144 | | 1.308 | 0.085 | 0.000 |  | 0.982 | 0.000 |
| 2nd | 290 | 22.40 | | 5.46 | 4.345 | | 45.233 | 0.124 | 0.000 |  | 0.753 | 0.000 |
| 3rd | 284 | 19.67 | | 4.15 | 0.765 | | 1.226 | 0.095 | 0.000 |  | 0.965 | 0.000 |
| IOP(SD) | 280 | 22.25 | | 3.48 | 1.684 | | 11.836 | 0.076 | 0.001 |  | 0.907 | 0.000 |
| Spherical equivalent (D) | 295 | 0.60 | | 10.73 | 4.778 | | 28.663 | 0.366 | 0.000 |  | 0.465 | 0.000 |
| Corneal radius of curvature (um) | 301 | 5.71 | | 0.22 | 0.252 | | 0.550 | 0.054 | 0.037 |  | 0.992 | 0.096 |
| Anterior chamber depth (um) | 307 | 3.25 | | 0.31 | -0.083 | | 2.290 | 0.074 | 0.000 |  | 0.967 | 0.000 |
| Lens thickness (um) | 307 | 3.41 | | 0.96 | 15.131 | | 252.110 | 0.278 | 0.000 |  | 0.216 | 0.000 |
| Axial length (um) | 307 | 18.76 | | 0.85 | 1.665 | | 2.661 | 0.187 | 0.000 |  | 0.807 | 0.000 |
| CCT (um) | 176 | 456.13 | | 32.91 | 0.365 | | -0.016 | 0.072 | 0.027 |  | 0.987 | 0.117 |
| CT (um) | 176 | 187.84 | | 38.34 | -0.585 | | 0.595 | 0.089 | 0.002 |  | 0.972 | 0.001 |
| Average RNFL thickness (um) | |  | | | |  | | | | | | |
| Superior  Inferior  Nasal  Temporal | 237  237  237  237 | 112.91  128.80  58.30  71.70 | | 14.61  15.45  12.731  14.100 | -0.150  -0.022  -0.260  0.165 | | 1.110  0.456  2.507  2.665 | 0.052  0.048  0.066  0.072 | 0.200  0.200  0.015  0.004 |  | 0.987  0.994  0.969  0.956 | 0.029  0.441  0.000  0.000 |

Note : the table is about **normality test of OD.** RNFL stands for retinal nerve fiber layer, ACD stands for Anterior chamber depth, Axial length stands for Axial length, CCT stands for Central corneal thickness, CT stands for Choroidal thickness

Supplementary table4. The ocular parameters of **normality test in OS**

|  | [sample size](javascript:;) | [mean](javascript:;) | | | | SD | [skewness](javascript:;) | | [kurtosis](javascript:;) | kolmogorov-smirnov test | |  | Shapiro-Wilk W test | |
| --- | --- | --- | --- | --- | --- | --- | --- | --- | --- | --- | --- | --- | --- | --- |
|  |  |  |  |  |  |  |  |  |  | statistic | P |  | statistic | P |
| IOP (mmHg) |  | | |  | | | | | | | | | | |
| 1st | 518 | | 23.24 | | 3.52 | | 0.294 | | 0.906 | 0.098 | 0.000 |  | 0.981 | 0.000 |
| 2nd | 291 | | 22.10 | | 4.42 | | 0.736 | | 3.745 | 0.078 | 0.000 |  | 0.960 | 0.000 |
| 3rd | 285 | | 19.22 | | 4.07 | | 0.573 | | 0.112 | 0.108 | 0.000 |  | 0.971 | 0.000 |
| IOP(SD) | 282 | | 21.80 | | 3.19 | | 0.306 | | 0.427 | 0.047 | 0.200 |  | 0.989 | 0.032 |
| Spherical equivalent (D) | 297 | | -0.32 | | 6.60 | | 4.41 | | 30.22 | 0.262 | 0.000 |  | 0.589 | 0.000 |
| Corneal radius of curvature (um) | 301 | | 5.70 | | 0.23 | | 0.104 | | 0.911 | 0.048 | 0.086 |  | 0.992 | 0.096 |
| Anterior chamber depth (um) | 308 | | 3.26 | | 0.30 | | -0.005 | | 1.177 | 0.066 | 0.003 |  | 0.976 | 0.000 |
| Lens thickness (um) | 308 | | 3.35 | | 0.28 | | 0.104 | | 1.347 | 0.052 | 0.041 |  | 0.980 | 0.000 |
| Axial length (um) | 308 | | 18.77 | | 0.88 | | 1.723 | | 2.653 | 0.191 | 0.000 |  | 0.792 | 0.000 |
| CCT (um) | 294 | | 459.08 | | 47.41 | | 4.586 | | 37.767 | 0.145 | 0.000 |  | 0.698 | 0.000 |
| CT (um) | 176 | | 190.16 | | 37.08 | | -0.578 | | 0.610 | 0.082 | 0.006 |  | 0.971 | 0.001 |
| Average RNFL thickness (um) | | |  | | | | |  | | | | | | |
| Superior  Inferior  Nasal  Temporal | 286  286  286  286 | | 111.85  128.20  56.36  75.31 | | 18.13  19.61  16.778  15.66 | | 4.060  0.514  4.467  1.339 | | 41.636  9.584  44.614  6.472 | 0.115  0.078  0.129  0.094 | 0.000  0.000  0.000  0.000 |  | 0.773  0.908  0.709  0.914 | 0.286  0.000  0.000  0.000 |

Note : the table is about **normality test of OS**. RNFL stands for retinal nerve fiber layer, ACD stands for Anterior chamber depth, Axial length stands for Axial length, CCT stands for Central corneal thickness, CT stands for Choroidal thickness

Supplementary Fig1. The flow chart of NHPES exclusion criteria and quality control (QC) process.


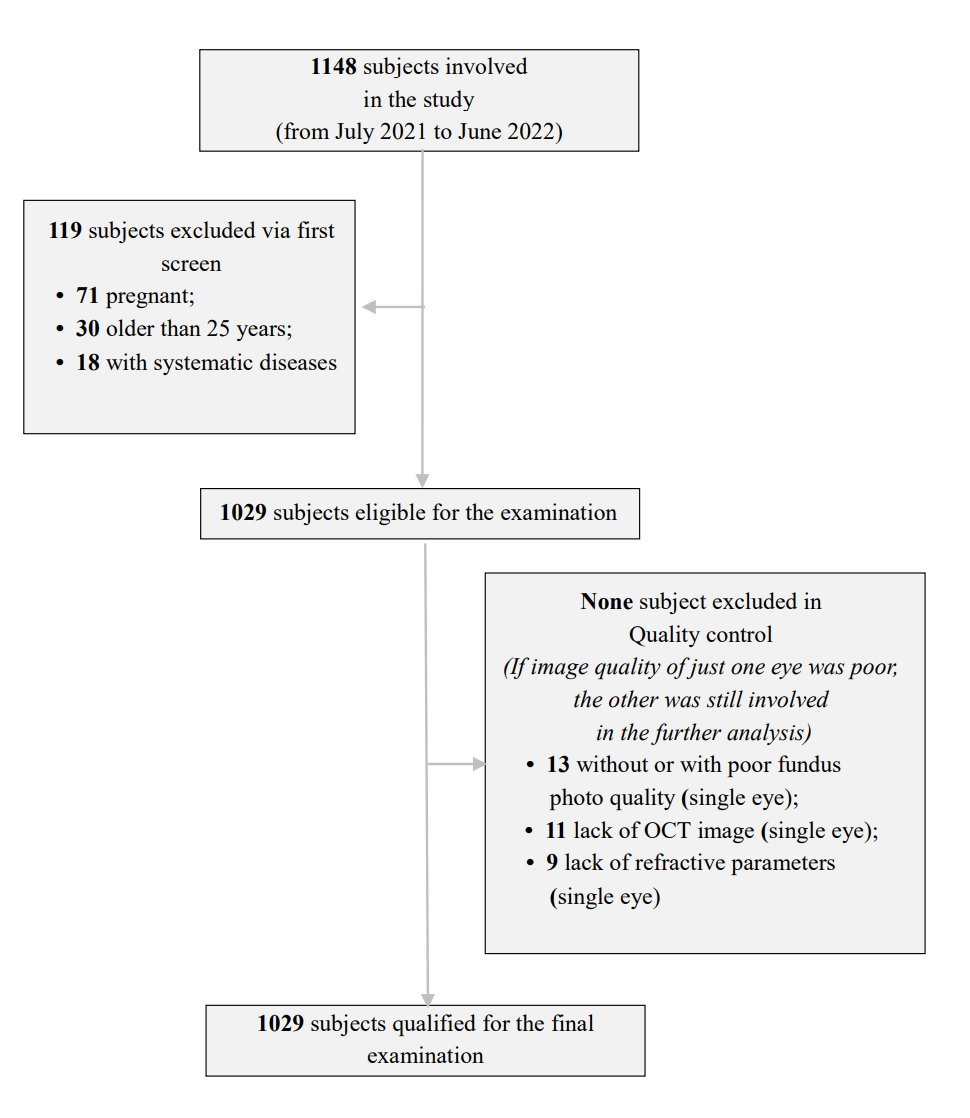

Supplement: Supplementary file 1 — Additional file 1: Supplementary Table 1. The prevalence of ocular diseases in NHPES [7, 13, 32–34]. Supplementary Table 2. The result of ocular parameters for gender in different age groups [35–46]. Supplementary Table 3. The ocular parameters of normality test in OD. Supplementary Table 4. The ocular parameters of normality test in OS. Supplementary Fig. 1. The flow chart of NHPES exclusion criteria and quality control (QC) process. [file 12886_2023_2796_MOESM1_ESM.docx]
