# Supplementary material for: Design, methodology, and preliminary results of the non-human primates eye study
Source: BMC Ophthalmol. 2023 Feb 7;23:53. doi: 10.1186/s12886-023-02796-6 (PMC9903517; doi:10.1186/s12886-023-02796-6)
Supplement: Supplementary file 2 — Additional file 2: Appendix. The non-human primates (NHP) eye study team. [file 12886_2023_2796_MOESM2_ESM.docx]

**APPENDIX: THE NON-HUMAN PRIMATES (NHP) EYE STUDY TEAM
Investigators**Yehong Zhuo, M.D., Ph.D., (Principal Investigator), Ningli Wang, M.D., Ph.D., (Co-Principal Investigator), Wenru Su, M.D., Ph.D., Jian Wu, M.D., Ph.D., Kezhe Chen, M.D., Wei Liu, Ph.D.

**Project manager**

Jian Wu, M.D., Ph.D.

**Study Coordinators**Sirui Zhu, M.D., Hongyi Liu, M.D., Chenlong Yang, M.D., Ph.D., Yingting Zhu, M.D., Ph.D., Zhidong Li, M.D., Ph.D., Lijie Pan, M.D., Ruyue Li, M.D., Caixia Lin, M.D., Ph.D., Jiaxin Tian, M.D., Ph.D.

**Study Examiners**Liangzhi Xu, Hanxiang Yu, Fagao Luo

**External Advisory Board Members**

Zhiwe Huang, Ph.D., Jiaoyan Ren, Ph.D.
